# Dissecting the functional divergence of DCAF11 isoforms in protein degradation

**DOI:** 10.1039/d6cb00119j

**Published:** 2026-07-10

**Authors:** Xiaokang Jin, Xiaoyu Zhang

**Affiliations:** a Department of Chemistry, Northwestern University Evanston Illinois 60208 USA zhang@northwestern.edu; b Chemistry of Life Processes Institute, Northwestern University Evanston Illinois 60208 USA; c Robert H. Lurie Comprehensive Cancer Center, Northwestern University Chicago Illinois 60611 USA; d Center for Human Immunobiology, Northwestern University Chicago Illinois 60611 USA; e International Institute for Nanotechnology, Northwestern University Evanston Illinois 60208 USA

## Abstract

DCAF11 is a substrate receptor of the Cullin-RING ligase 4 (CRL4) ubiquitin ligase complex and an emerging effector supporting targeted protein degradation. DCAF11 exists as two major isoforms, but their functional differences remain incompletely understood. Here, we show that DCAF11 isoforms 1 and 2 assemble into CRL4 complexes with similar efficiency and exhibit largely overlapping endogenous substrate profiles, including proteins implicated in electrophile detoxification. In contrast, they differ in their compatibility with small-molecule degraders: covalent PROTACs engage both isoforms, whereas a non-covalent molecular glue selectively utilizes isoform 1. These findings reveal isoform-dependent control of protein degradation and highlight opportunities for isoform-selective targeting.

## Abstract 2

DCAF11 isoforms share similar endogenous degradation profiles but differ in induced protein degradation, with covalent PROTACs recruiting both isoforms and a molecular glue selectively engaging isoform 1.

## Graphical abstract

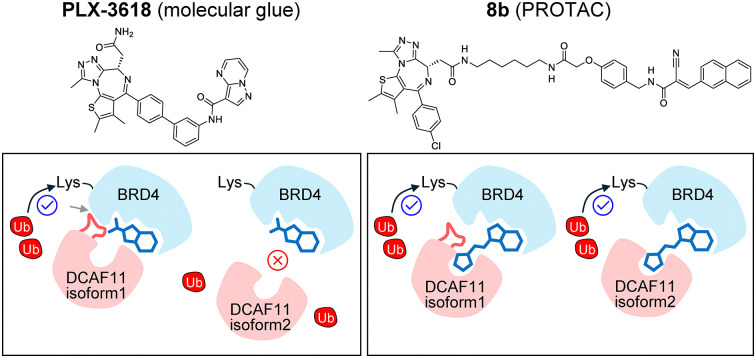

## Introduction

Cullin-RING ligases (CRLs) are central to protein homeostasis,^1,2^ with substrate specificity conferred by interchangeable adaptor proteins that recognize defined degron motifs.^3,4^ DCAF11 (also known as WDR23) functions as a substrate receptor within the CRL4-DDB1 complex and has emerged as an important regulator of protein stability in stress-responsive pathways.^5,6^ One of its characterized roles is in controlling the turnover of the transcription factor NRF2, a master regulator of antioxidant and xenobiotic stress responses.^6^ In addition to the canonical KEAP1-CUL3 pathway, DCAF11 provides an alternative route for NRF2 ubiquitination and degradation, thereby contributing to multilayered regulation of NRF2 activity and cellular redox homeostasis.^6^ Despite this established function, the broader physiological roles, substrate scope, and regulatory mechanisms of DCAF11 remain incompletely defined.

More recently, DCAF11 has gained attention as a ligase that can be co-opted for targeted protein degradation (TPD), where small molecules, including both heterobifunctional Proteolysis-Targeting Chimeras (PROTACs) and monofunctional molecular glues, recruit DCAF11 to induce proximity-driven ubiquitination of neo-substrates.^7–17^ DCAF11 exists as two major isoforms that differ in sequence and subcellular localization, with isoform 1 primarily cytoplasmic and isoform 2 enriched in the nucleus.^6^ While both isoforms have been implicated in NRF2 regulation, whether they exhibit distinct degradation profiles toward other endogenous substrates or in ligand-induced degradation remains unclear. In this study, we dissect the contributions of DCAF11 isoforms to both native and ligand-induced protein degradation, providing mechanistic insight into isoform-dependent regulation of CRL4-DCAF11 activity.

## Experimental

### Reagents

The HRP-linked anti-HSP90 (clone #C45G5, cat. #79641, dilution 1 : 5000), anti-HA (clone #C29F4, cat. #3724, dilution 1 : 5000), HRP-linked anti-rabbit IgG (cat. #7074, dilution 1 : 1000), anti-GAPDH (clone #14C10, cat. #3683, dilution 1 : 1000), anti-Lamin A/C (polyclonal, cat. #2032, dilution 1 : 1000), and anti-BRD4 (clone #E2A7X, cat. #13440, dilution 1 : 1000) antibodies were purchased from Cell Signaling Technology. The anti-*β*-Actin HRP antibody (clone #C4, cat. #sc-47778, dilution 1 : 1000) was purchased from Santa Cruz Biotechnology. The anti-FLAG HRP-conjugated antibody (clone M2, cat. #A8592, dilution 1 : 5000) and anti-HA agarose (cat. #A2095) were purchased from Sigma-Aldrich. The anti-DCAF11 antibody (cat. #A15519, dilution 1 : 1000) was purchased from Abclonal. FuGene 6 (cat. #E2692) transfection reagent, Cell Titer Glo (cat. #G7573) and sequencing grade trypsin (cat. # V5111) and Glu-C (cat. #V1651) were purchased from Promega. Polyethylenimine (molecular weight 40 000, cat. #24765-1) was purchased from Polysciences. DC protein assay kit (cat. #5000112) was purchased from Bio-Rad. Pierce high pH reversed-phase peptide fractionation kit (cat. #84868), Pierce ECL Western Blotting substrate (cat. #32209), SuperSignal West Pico PLUS chemiluminescent substrate (cat. #34580), subcellular protein fractionation kit (cat. # 78840), and Tandem Mass Tag (TMT) isobaric label reagent (cat. #90110) were purchased from Thermo Scientific. PLX-3618 (cat. #HY-161779), 8b (cat. #HY-158764), 4-Hydroxynonenal (cat. #HY-113466), and (1*S*,3*R*)-RSL3 (cat. #HY-100218A) were purchased from MedChemExpress.

### Cell Lines

22Rv1 cells were obtained from ATCC and cultured in RPMI-1640 medium (Corning, cat. #15040CV) with 10% (v/v) fetal bovine serum (FBS, Omega Scientific, cat. #FB-01) and l-glutamine (2 mM, Gibco, cat. #25030081). HEK293T cells were obtained from ATCC and cultured in Dulbecco's Modified Eagle Medium (DMEM, Corning, cat# 15013CV) with 10% (v/v) fetal bovine serum (FBS, Omega Scientific, cat. #FB-01) and l-glutamine (2 mM, Gibco, cat. #25030081). All cell lines were tested negative for mycoplasma contamination using Universal Mycoplasma Detection Kit (ATCC, cat. #30-1012K).

### Subcellular Fractionation

Cells were collected and fractionated using the Subcellular Protein Fractionation Kit (Thermo Scientific, Cat. #78840) according to the manufacturer's instructions. Briefly, 10^6^ cells were lysed in 100 µL of Cytoplasmic Extraction Buffer and incubated on ice for 10 minutes. The lysate was centrifuged at 500 g for 5 minutes, and the supernatant was collected as the cytoplasmic fraction. The pellet was resuspended in 100 µL of Membrane Extraction Buffer, vortexed at high speed for 5 seconds, and incubated on ice for 10 minutes. Following centrifugation at 3000 g for 5 minutes, the supernatant was collected as the membrane fraction. The remaining pellet was resuspended in 50 µL of nuclear extraction buffer, vortexed for 15 seconds, and incubated on a shaker at 4 °C for 30 minutes. After centrifugation at 5000 g for 5 minutes, the supernatant was collected as the soluble nuclear fraction. The pellet was resuspended in 50 µL of nuclear extraction buffer supplemented with 2.5 µL of 100 mM CaCl_2_ and 1.5 µL of Micrococcal Nuclease. The mixture was vortexed for 15 seconds, incubated at room temperature for 15 minutes, and centrifuged at 16000 g for 5 minutes. The supernatant was collected as the chromatin-bound nuclear fraction. The remaining pellet was resuspended in 50 µL of pellet extraction buffer, vortexed for 15 seconds, incubated at room temperature for 10 minutes, and centrifuged at 16000 g for 5 minutes. The supernatant was collected as the cytoskeletal fraction. All fractions were mixed with Laemmli sample buffer and heated at 95 °C for 5 minutes to denature proteins prior to western blot analysis.

### Cell viability assay

22Rv1 cells were seeded into the 96-well plate (Corning, cat. #3610) at a density of 10 000 cells per well in 100 µL of medium. The cells were treated with serial dilutions of compounds in 100 µL of medium for 72 hours. 50 µL of Cell Titer Glo reagent was added to each well and incubated for 10 minutes at room temperature. Luminescence was quantified using a BMG CLARIOstar Plus microplate reader.

### Cloning and lentiviral transduction

Expression constructs encoding N-terminal HA-tagged human DCAF11 isoform 1 or isoform 2 in the pCDH-CMV-MCS-EF1-Puro vector were sourced from GenScript. Lentivirus was generated by co-transfecting HEK293T cells with the expression construct with psPAX2 and pMD2.G plasmids using FuGene 6. Viral supernatants were collected 48 hours after transfection, passed through 0.45 µm Millex-HV sterile syringe filters (MilliporeSigma), and used to infect 22Rv1 cells. Transduced cells were selected with puromycin (2 µg mL^−1^) for 5 days to establish stable polyclonal populations.

### Transient Transfection

Expression constructs encoding N-terminal FLAG-tagged human FKBP12 with or without a C-terminal PKKKRKV nuclear localizing sequence in the pCDH-CMV-MCS-EF1-Puro vector were sourced from GenScript. A plasmid encoding FLAG-BRD4 in the pRK5 vector was used for co-immunoprecipitation. Cells were seeded to 50% confluency in 6-well plates or 10 cm dishes. Total of 2 µg (6-well plate) or 6 µg (10-cm dish) of plasmid DNA was diluted in serum free RPMI-1640 medium with 6 µL or 18 µL of PEI were added, respectively. The mixture was incubated at room temperature for 15 minutes and added dropwise to the cells. Cells were grown for 24 hours after transfection, and then were split for treatment followed by western blots or co-immunoprecipitation.

### Western blot

Cells were lysed in RIPA buffer containing 25 mM Tris-HCl (pH 7.6), 150 mM NaCl, 1% NP-40, 1% sodium deoxycholate, and 0.1% SDS, supplemented with universal nuclease immediately before use. Protein concentrations were measured using the DC protein assay. Lysates were combined with Laemmli sample buffer and heated at 95 °C for 5 minutes to denature proteins. Samples were resolved on either 4–20% or 8% Novex Tris-Glycine gels, followed by transfer onto 0.2 µm PVDF membranes (Bio-Rad, cat. #1620177). Membranes were blocked in TBST buffer (20 mM Tris-HCl, pH 7.6, 150 mM NaCl, 0.1% Tween-20) containing 5% non-fat milk for 1 hour at room temperature. Primary antibodies diluted in blocking buffer were incubated overnight at 4 °C. After three washes with TBST, membranes were incubated with secondary antibodies for 1 hour at room temperature, followed by additional washes. Signals were developed using ECL chemiluminescent reagents and detected on a Bio-Rad ChemiDoc MP imaging system.

### Global proteomics

Cells were lysed in 100 µL PBS supplemented with cOmplete protease inhibitor cocktail, followed by sonication (five pulses at 40% amplitude, repeated over three cycles). Protein concentrations were determined using the DC protein assay. For denaturation, 100 µg of total protein in 50 µL PBS was combined with an equal volume of 12 M urea prepared in PBS. Disulfide bonds were reduced by adding 5 µL of 200 mM DTT and incubating at 65 °C for 15 minutes, followed by alkylation with 5 µL of 400 mM iodoacetamide for 30 minutes at 37 °C in the dark. Samples were then diluted with 300 µL PBS and digested with 2 µg trypsin/LysC at 37 °C for 12 hours. For tandem mass tag (TMT) labeling, 8.5 µg of peptides were resuspended in 35 µL PBS, supplemented with 9 µL acetonitrile, and incubated with TMT reagents (5 µL per channel) for 1 hour at room temperature. Reactions were quenched by sequential addition of 6 µL of 5% hydroxylamine and 2.5 µL formic acid. Labeled samples were pooled and fractionated into ten fractions using a high-pH reversed-phase peptide fractionation kit. Fractions were dried by vacuum centrifugation and subjected to LC–MS analysis on an Orbitrap Eclipse Tribrid mass spectrometer coupled to a Vanquish Neo UHPLC system. Peptides were separated on an EASY-Spray C18 column (2 µm particle size, 75 µm inner diameter, 150 mm length) at a flow rate of 0.25 µL min^−1^ using a multistep gradient. Mobile phase A consisted of 0.1% formic acid in water, and mobile phase B consisted of 80% acetonitrile with 0.1% formic acid. The gradient was maintained at 5% B for 0–15 minutes, increased from 5% to 35% B over 15–160 minutes, and then ramped to 100% B from 160–180 minutes. Nanoelectrospray ionization was operated at 1.5 kV. Full MS scans were acquired in the Orbitrap (resolution 60 000; *m/z* 375–1600; RF lens 60%; standard AGC; automatic maximum injection time). Precursor ions were isolated with a 0.7 *m/z* window and fragmented by HCD in the ion trap for MS2 analysis (collision energy 27%; standard AGC; maximum injection time 35 ms). For MS3, up to ten fragment ions were selected *via* synchronous precursor selection, further fragmented by HCD, and analyzed in the Orbitrap (collision energy 55%; AGC target 250%; maximum injection time 200 ms; resolution 60 000). Data acquisition was performed using Xcalibur software (v4.5.445.18, Thermo Scientific). Raw data were processed using Proteome Discoverer (v2.5, Thermo Scientific). Peptide identification was carried out with the Sequest HT search engine against the UniProt human reference proteome (UP000005640_9606_Human.fasta).

### Affinity purification mass spectrometry (AP–MS)

Cells were lysed in NP-40 buffer composed of 25 mM Tris–HCl (pH 7.6), 150 mM NaCl, 10% glycerol, and 1% NP-40, supplemented with complete protease inhibitor cocktail. Lysates were clarified by centrifugation at 16 000 g for 10 minutes at 4 °C, and the supernatants were used for immunoprecipitation. HA affinity agarose (30 µL slurry per sample) was incubated with the lysates under rotation for 2 hours at 4 °C. Beads were washed four times with immunoprecipitation wash buffer, followed by an additional wash with PBS. Proteins bound to the resin were eluted by heating at 65 °C for 10 minutes in 8 M urea prepared in PBS. Eluted samples were reduced with 12.5 mM DTT at 65 °C for 15 minutes and then alkylated with 25 mM iodoacetamide at 37 °C for 30 minutes. The urea concentration was subsequently decreased to 2 M by dilution with PBS prior to proteolytic digestion with 2 µg trypsin/LysC at 37 °C for 12 hours. TMT labeling was performed by adding 6 µL of reagent (in anhydrous acetonitrile) and incubating for 1 hour at room temperature. Labeling reactions were quenched with 6 µL of 5% hydroxylamine followed by 2.5 µL of formic acid. Peptide samples were pooled, desalted using a Sep-Pak C18 cartridge (Waters, cat. #WAT054955), and dried under vacuum. Processed samples were then subjected to LC–MS analysis as described above, and data analysis was carried out using the same database search and quantification parameters.

### Statistical analysis

Quantitative measurements are shown as scatter plots, with error bars indicating the mean ± standard error of the mean (SEM). For proteomics datasets, statistical significance was assessed using two-tailed Student's *t*-tests, and the resulting *P* values were adjusted for multiple hypothesis testing using the Benjamini–Hochberg procedure.

## Results and discussion

### DCAF11 isoforms 1 and 2 exhibit similar CRL assembly

**Fig. 1:**
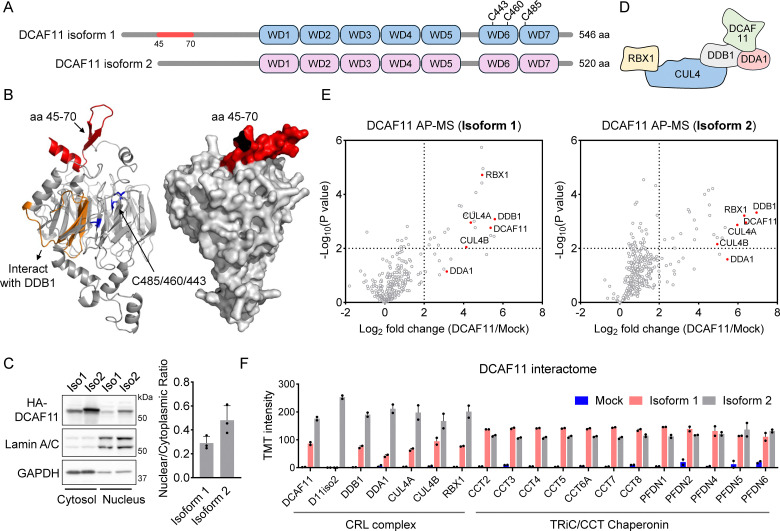
DCAF11 isoforms 1 and 2 exhibit similar CRL assembly. (A) Schematic of DCAF11 isoforms 1 and 2 highlighting the sequence difference (residues 45–70 present in isoform 1 and absent in isoform 2) and the conserved WD40 repeat domains. (B) AlphaFold2-predicted structure of DCAF11 isoform 1 showing the low-confidence sequence region (aa 45–70, highlighted in red) and the well-folded WD40 *β*-propeller core. Key cysteine residues (C443, C460, and C485) implicated in covalent DCAF11-based PROTAC engagement are indicated. (C) Subcellular fractionation analysis of DCAF11 isoforms 1 and 2 in 22Rv1 cells. The bar graph shows the quantification of the nuclear-to-cytoplasmic ratio of DCAF11 protein levels. Data are presented as mean ± SEM (*n*  =  3 independent replicates). Iso, isoform. (D) Schematic of the CUL4-based CRL complex containing DCAF11, DDB1, DDA1, CUL4, and RBX1. (E) Volcano plot of AP–MS data for DCAF11 isoforms 1 and 2 (*n* = 2 biologically independent samples). (F) Quantification of AP–MS enrichment for CRL components and TRiC/CCT chaperonin proteins (*n* = 2 biologically independent samples).

DCAF11 exists as two major isoforms that differ in sequences near the N-terminus, with residues 45–70 present in isoform 1 but absent in isoform 2, resulting in proteins of distinct lengths (∼546 aa and ∼520 aa, respectively) (Fig. 1A). As no experimentally determined structure of DCAF11 has been reported to date, we used the AlphaFold2-predicted model of isoform 1 to examine this sequence difference. The model reveals that the isoform-specific region (aa 45–70) has low predicted confidence, whereas the remaining regions, including the WD40 domain implicated in DDB1 and substrate interaction and the three cysteine residues (C443, C460, and C485) involved in covalent DCAF11-based PROTACs,^12,14,15^ exhibit high-confidence structural features consistent with a well-folded core architecture (Fig. 1B).

To study the two DCAF11 isoforms, we used our previously reported 22Rv1 parental and DCAF11 knockout (KO) cells, in which DCAF11 has been established as a functional E3 ligase,^15^ and reintroduced either DCAF11 isoform 1 or isoform 2. Mass spectrometry analysis identified isoform 2 specific peptides from both trypsin and Glu-C digestion in DCAF11 isoform 2 expressing cells, but not in isoform 1 expressing cells (Fig. S1A and B), confirming that isoform 2 is expressed as expected. Next, we examined the subcellular localization of both isoforms. Consistent with previous findings,^6^ both isoforms were detected in the cytoplasm and nucleus, with the cytoplasm representing the predominant compartment for each. Notably, isoform 2 exhibited a higher nuclear-to-cytoplasmic ratio than isoform 1, indicating greater relative nuclear enrichment (Fig. 1C).

DCAF11 contains a conserved WDxR DDB1-binding motif and forms a complex with DDB1, DDA1, CUL4, and RBX1, functioning as the substrate receptor of a CUL4-based CRL complex^18^ (Fig. 1D). To determine whether isoform 2 can be incorporated into this complex similarly to isoform 1, we performed affinity purification coupled with mass spectrometry (AP–MS). The results show that both isoforms assemble into the CRL complex, as each interacts with these core components to a similar extent after normalization to DCAF11 levels (Fig. 1E and F and Table S1). Interestingly, the proteomic data suggest that isoform 1 may interact more strongly with multiple components of the TCP-1 ring complex (TRiC), also known as the chaperonin containing TCP-1 (CCT), than isoform 2 (Fig. 1F). Given that the TRiC/CCT chaperonin complex is involved in the folding of structurally complex proteins,^19^ these findings raise the possibility that isoform 1 and isoform 2 may differ in their folding dynamics. Such differences could potentially arise from the N-terminal extension unique to isoform 1. Additional biochemical studies will be required to validate the observed interactions and determine their functional significance.

### DCAF11 isoforms 1 and 2 regulate a shared substrate network implicated in electrophile detoxification

**Fig. 2:**
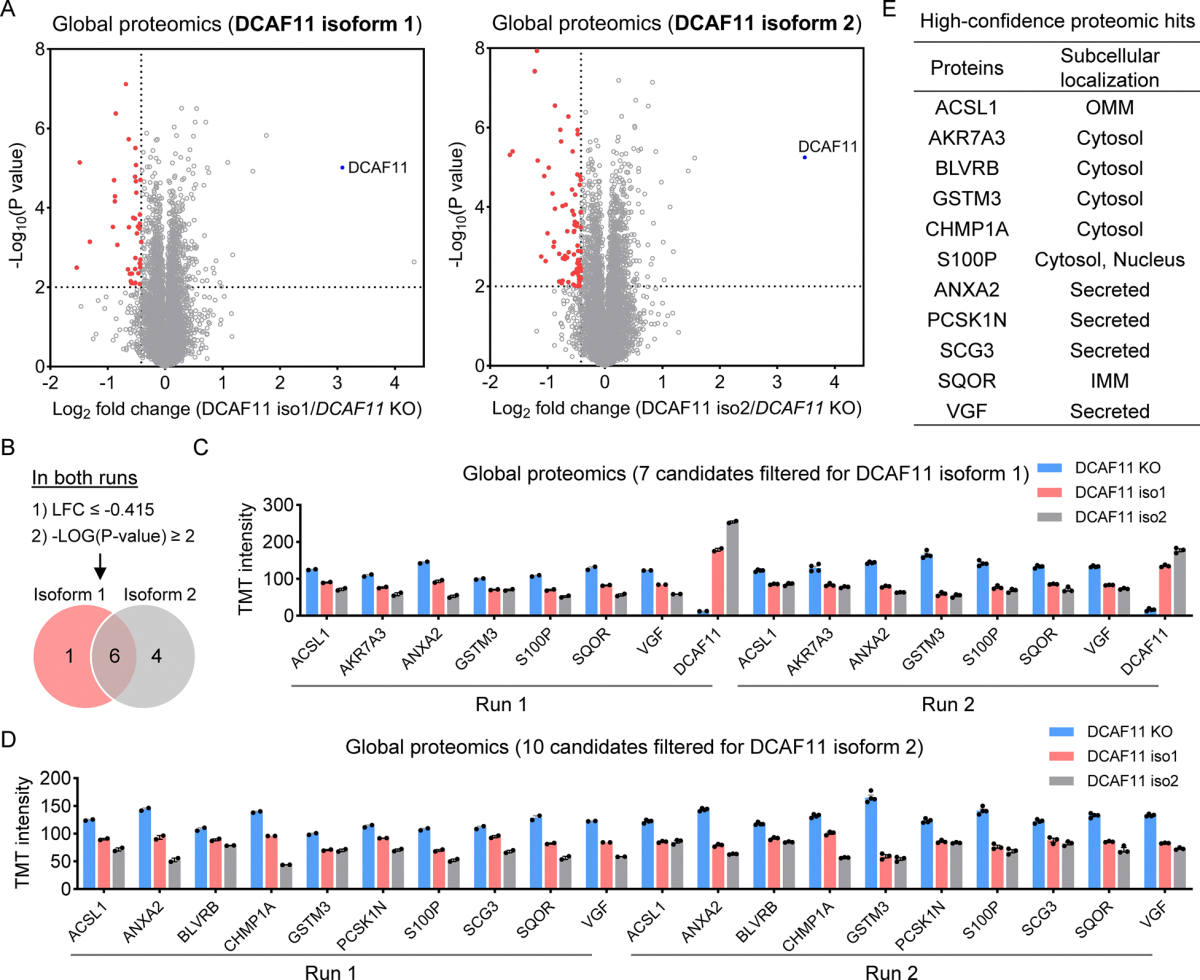
DCAF11 isoforms 1 and 2 regulate a shared substrate network. (A) Volcano plot of global proteomics data comparing DCAF11 KO cells with KO cells re-expressing DCAF11 isoforms 1 and 2 (*n* = 3 biologically independent samples). *P* values were calculated using a two-sided *t*-test and adjusted for multiple comparisons by the Benjamini–Hochberg method. (B) Venn diagram showing overlap of high-confidence downregulated proteins identified in isoform 1 and isoform 2 datasets based on defined statistical thresholds. (C) Quantification of candidate proteins demonstrating similar trends of reduction across both isoforms, based on filtering criteria applied to DCAF11 isoform 1. (D) Quantification of candidate proteins demonstrating similar trends of reduction across both isoforms, based on filtering criteria applied to DCAF11 isoform 2. (E) Subcellular localization annotation of identified candidate substrates.

To explore whether the two DCAF11 isoforms exhibit distinct degradation profiles toward endogenous protein substrates, we performed global proteomic analysis comparing 22Rv1 DCAF11 KO cells with KO cells re-expressing either isoform 1 or isoform 2. This analysis identified several proteins that were downregulated upon DCAF11 re-expression (Fig. 2A and Table S2). Our global proteomics analysis was performed using a multiplexed format that combined three biologically independent samples, generating the dataset shown in Fig. 2A. To increase confidence in hit identification, we conducted an additional global proteomics experiment using a similar multiplexing strategy (Fig. S2). For stringent data filtering, proteins were required to meet the following criteria in both datasets: log_2_ fold change (LFC; isoform-expressing *versus* DCAF11 KO) ≤ −0.415 (≥ 25% reduction in protein abundance) and -log_10_ (*P*-value) ≥ 2 (*P* value ≤ 0.01). Applying these filters yielded 7 high-confidence hits in isoform 1-expressing cells and 10 high-confidence hits in isoform 2-expressing cells, with 6 proteins overlapping between the two isoforms (Fig. 2B). We further examined proteins that did not overlap between isoforms and found that these candidates exhibited similar trends of reduction in the alternate isoform condition but fell just below the defined thresholds (Fig. 2C and D). This suggests that the apparent differences between isoforms are likely due to threshold stringency rather than true isoform-specific activity. Overall, these results indicate that DCAF11 isoforms 1 and 2 may drive largely similar protein reduction profiles in 22Rv1 cells.

Among the 11 total candidate substrates (Fig. 2E), five proteins are annotated as secreted or localized to the inner mitochondrial membrane (IMM), suggesting they are unlikely to directly interact with DCAF11 and may instead be regulated through indirect mechanisms. In contrast, the remaining six proteins, many of which have not been previously reported to be regulated by DCAF11, are localized to the cytosol, nucleus, or outer mitochondrial membrane (OMM), placing them in cellular compartments where they could potentially interact directly with DCAF11 (Fig. 2E). Several of these proteins, including ACSL1, AKR7A3, BLVRB, and GSTM3, are well-established NRF2 target gene products.^20,21^ Their increased abundance in DCAF11 KO cells may therefore reflect downstream consequences of NRF2 stabilization following DCAF11 loss, consistent with the reported role of DCAF11 as an alternative E3 ligase for NRF2.^6^ However, NRF2 was not detected in our proteomic dataset (Table S2), likely due to its relatively low abundance, and therefore this possibility could not be directly evaluated. At the same time, we cannot exclude the possibility that some of these proteins may also be directly regulated by DCAF11. Additional studies will be required to distinguish between these possibilities.

**Fig. 3:**
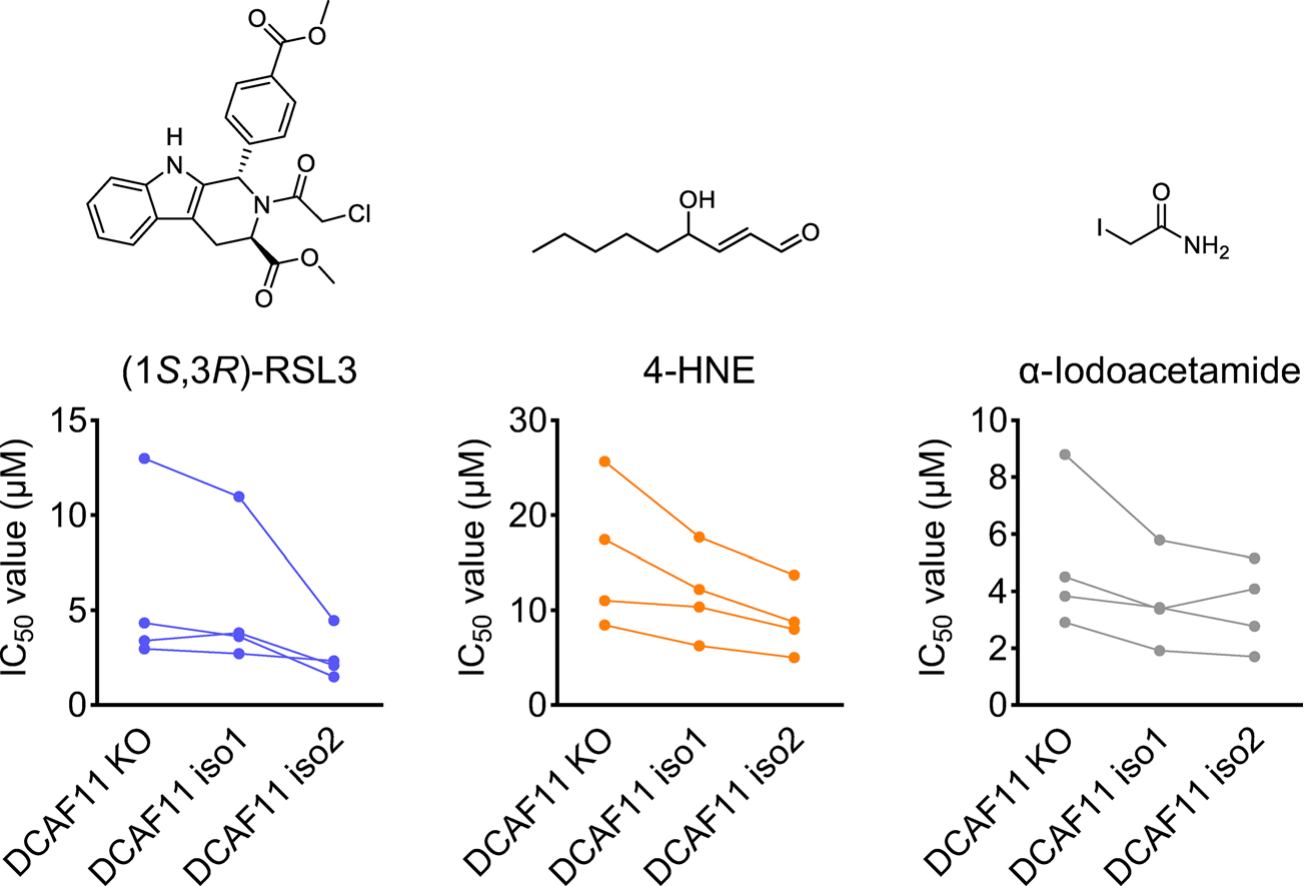
DCAF11 expression sensitizes cells to electrophilic agents. Top panel: chemical structures of (1*S*,3*R*)-RSL3, 4-hydroxynonenal (4-HNE) and *α*-iodoacetamide. Bottom panel: cytotoxic IC_50_ values of the indicated compounds in DCAF11 KO cells and KO cells re-expressing isoform 1 or isoform 2. Each point represents an individual biological replicate (*n* = 4), with lines connecting paired measurements from the same experiment.

Notably, ACSL1, AKR7A3, BLVRB, and GSTM3, are functionally linked to detoxification pathways.^22,23^ In particular, AKR7A3 and GSTM3 play key roles in the detoxification of electrophilic species. These observations suggest a potential role for DCAF11 in modulating cellular responses to electrophilic stress. To test this hypothesis, we treated cells with representative electrophiles, including 4-hydroxynonenal (4-HNE), (1*S*,3*R*)-RSL3, and *α*-iodoacetamide. Re-expression of DCAF11 in DCAF11 KO cells modestly sensitized them to these electrophilic agents, with isoform 2 showing a slightly stronger effect (Fig. 3 and Fig. S3).

### DCAF11 isoforms 1 and 2 differentially support PROTAC- and molecular glue-mediated protein degradation

**Fig. 4:**
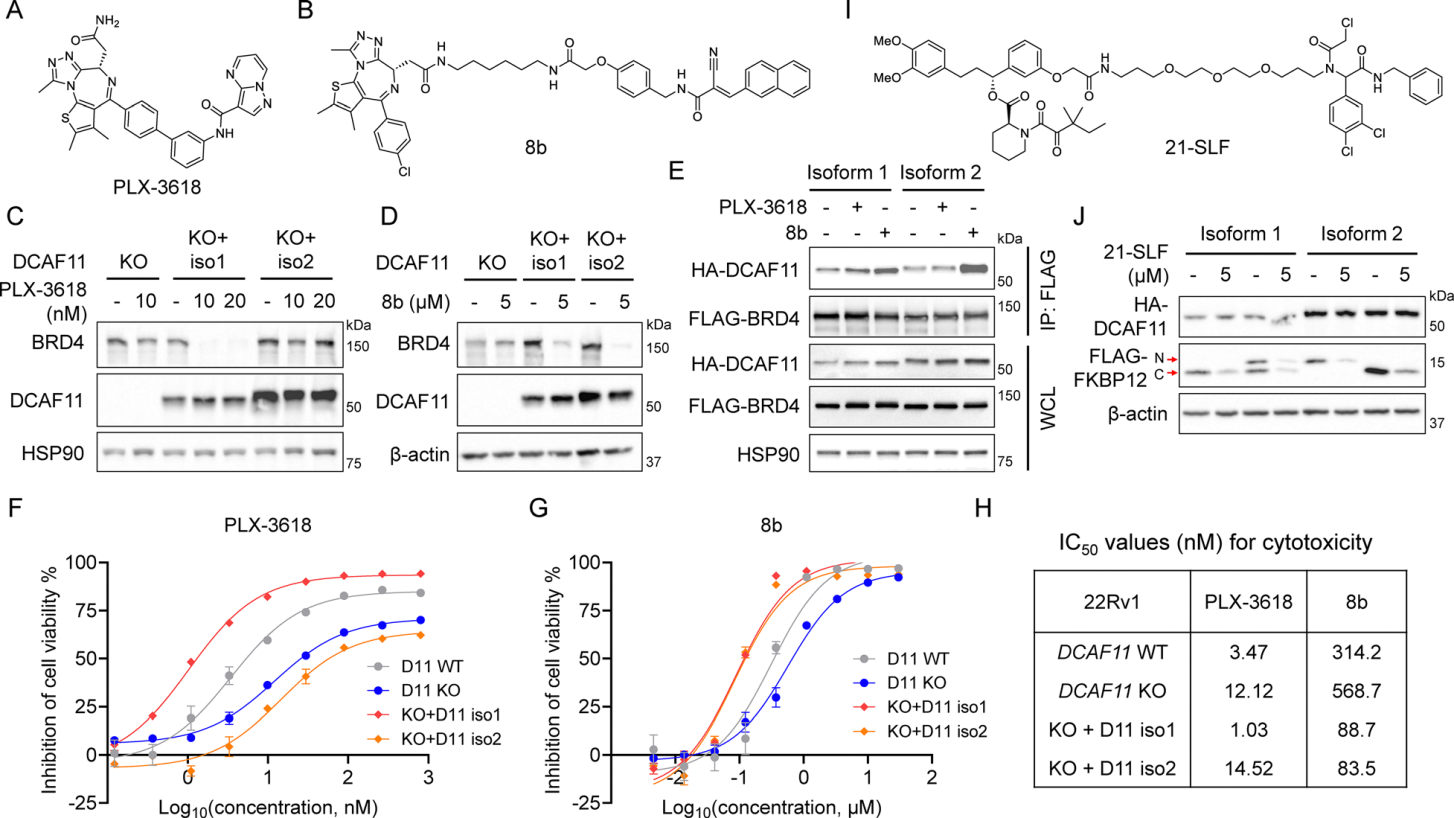
DCAF11 isoforms 1 and 2 differentially support PROTAC- and molecular glue-mediated protein degradation. (A) Chemical structure of PLX-3618. (B) Chemical structure of 8b. (C) Western blot analysis of BRD4 levels in DCAF11 KO cells and KO cells re-expressing DCAF11 isoform 1 or isoform 2 following treatment with PLX-3618 for 8 h. The result is representative of two experiments (*n* = 2 biologically independent samples). (D) Western blot analysis of BRD4 levels in DCAF11 KO cells and KO cells re-expressing DCAF11 isoform 1 or isoform 2 following treatment with 8b for 5 h. The result is representative of two experiments (*n* = 2 biologically independent samples). (E) Co-immunoprecipitation analysis of ternary complex formation between HA-DCAF11 isoform 1 or isoform 2 and FLAG-BRD4 in the presence of PLX-3618 (1 µM, 4 h) or 8b (1 µM, 4 h). All samples were pretreated with bortezomib (1 µM, 2 h) prior to compound treatment. The result is representative of two experiments (*n* = 2 biologically independent samples). Quantification of immunoprecipitated HA-DCAF11 is presented in Fig. S4. WCL, whole cell lysate. (F) Dose–response curves for cell viability following treatment with PLX-3618 in WT, DCAF11 KO, and isoform-expressed cells for 72 h. Data are presented as mean ± SEM (*n* = 3 biologically independent samples). (G) Dose–response curves for cell viability following treatment with 8b in WT, DCAF11 KO, and isoform-expressed cells for 72 h. Data are presented as mean ± SEM (*n* = 3 biologically independent samples). (H) Quantification of cytotoxic IC_50_ values corresponding to (F) and (G). (I) Chemical structure of 21-SLF. (J) Western blot analysis of FKBP12 levels in DCAF11 KO cells re-expressing DCAF11 isoform 1 or isoform 2 following treatment with 21-SLF for 4 h. The result is representative of two experiments (*n* = 2 biologically independent samples). N, nucleus-localized FKBP12; C, cytosolic FKBP12.

We next investigated whether DCAF11 isoforms 1 and 2 differ in their ability to support ligand-induced protein degradation. To this end, we evaluated two reported DCAF11-recruiting BRD4 degraders with distinct mechanisms: PLX-3618,^10^ a non-covalent degrader lacking a linker and resembling a molecular glue (Fig. 4A), and 8b,^12^ a covalent heterobifunctional PROTAC known to modify DCAF11 at C443/460/485 (Fig. 4B). Using western blot analysis, we first assessed BRD4 degradation in DCAF11 KO cells and KO cells re-expressing either isoform 1 or isoform 2. Neither compound induced BRD4 degradation in DCAF11 KO cells, confirming that their activity is dependent on DCAF11 (Fig. 4C and D). Re-expression of isoform 1 restored BRD4 degradation by both PLX-3618 and 8b, whereas isoform 2 supported BRD4 degradation by 8b but not by PLX-3618, indicating isoform-selective activity of the non-covalent molecular glue degrader (Fig. 4C and D).

To assess compound-induced ternary complex formation, we performed co-immunoprecipitation experiments in DCAF11 KO cells stably expressing either DCAF11 isoform 1 or isoform 2 and treated with DMSO, PLX-3618, or 8b. BRD4 was immunoprecipitated to evaluate its association with DCAF11 in the presence of each compound. Compared to the DMSO control, the heterobifunctional degrader 8b effectively induced ternary complex formation with both DCAF11 isoforms 1 and 2 (Fig. 4E and Fig. S4). In contrast, the molecular glue PLX-3618 induced only modest ternary complex formation with DCAF11 isoform 1 and no ternary complex formation with isoform 2 (Fig. 4E and Fig. S4). These findings are consistent with the degradation data, in which PLX-3618 induced BRD4 degradation only in cells expressing DCAF11 isoform 1, whereas 8b promoted BRD4 degradation in cells expressing either isoform.

Given that BRD4 is an essential protein,^24^ we next examined whether these differences in degradation translate into functional outcomes. Consistent with their dependence on DCAF11, both compounds exhibited reduced cytotoxicity in DCAF11 KO cells compared to WT cells (Fig. 4F–H). Re-expression of isoform 1 restored the cytotoxic effects of both degraders, whereas isoform 2 selectively rescued the activity of 8b but not PLX-3618 (Fig. 4F–H). Together, these results demonstrate that PLX-3618 may selectively engage DCAF11 isoform 1, whereas the covalent PROTAC 8b can utilize both isoforms to drive target degradation and associated functional outcomes. In agreement with these findings, our previously reported covalent DCAF11-based FKBP12 degrader, 21-SLF^15^ (Fig. 4I), induced efficient degradation of both nuclear and cytosolic FLAG-FKBP12 in cells expressing either isoform (Fig. 4J, note that the nuclear FKBP12 construct contains an additional nuclear localization sequence,^25^ PKKKRKV, which results in an electrophoretic mobility shift).

**Fig. 5:**
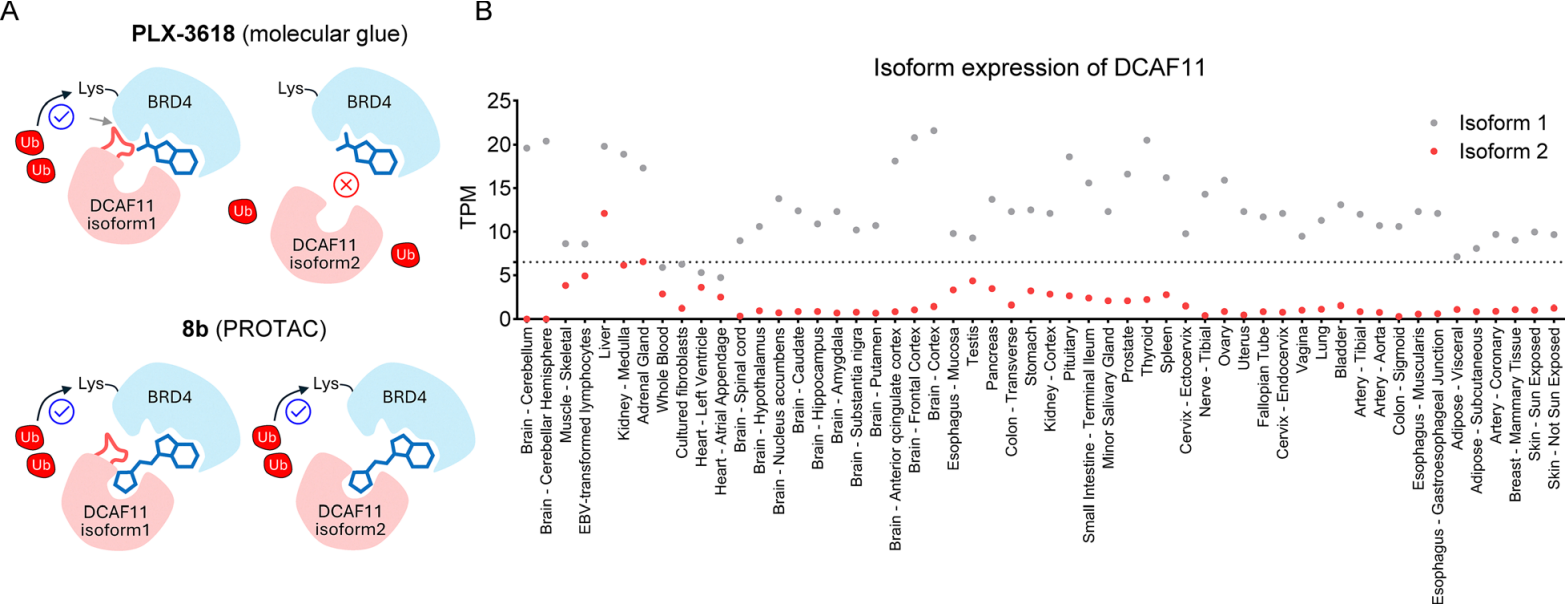
A model for isoform-selective DCAF11 engagement and tissue expression of DCAF11 isoforms. (A) Proposed model illustrating isoform-dependent engagement of DCAF11 by distinct degrader classes. (B) Gene expression of DCAF11 isoforms 1 and 2 across human tissues, based on data from the Genotype-Tissue Expression (GTEx) project. TPM, transcripts per million.

The isoform selectivity of PLX-3618 suggests that the sequence unique to isoform 1 (aa 45–70) may play a critical role in supporting PLX-3618-mediated BRD4 degradation. In contrast, covalent PROTACs are known to engage DCAF11 through C443/460/485,^12,15^ residues that are distal to the isoform 1-specific sequence and conserved between both isoforms. Consequently, these covalent degraders can induce target degradation by recruiting either DCAF11 isoform. Based on this, we propose a model in which structural differences between the isoforms influence their compatibility with distinct classes of degraders (Fig. 5A). Although our findings are currently limited to PLX-3618, they suggest that DCAF11 isoform expression may represent an underappreciated determinant of molecular glue activity. Future studies evaluating additional DCAF11 molecular glues with diverse chemical scaffolds will be important to determine whether the isoform selectivity observed here reflects a general feature of DCAF11-mediated molecular glue recruitment or is specific to the unique binding mode of PLX-3618.

In addition, our attempts to perform structural modeling are currently limited by the lack of an experimentally determined DCAF11 structure and the low confidence of the AlphaFold2 prediction for the isoform 1-specific insertion (aa 45–70). As a result, the structural basis underlying the isoform selectivity of PLX-3618 remains unclear. Future structural studies will be required to determine how the isoform 1-specific sequence contributes to PLX-3618-mediated BRD4 degradation. Notably, while isoform 1 is broadly expressed across tissues, isoform 2 exhibits enriched expression in the liver (Fig. 5B). This raises the possibility that DCAF11 isoform-selective degraders could be leveraged to achieve tissue-selective protein degradation. Future studies will be needed to determine whether endogenous DCAF11 isoform expression influences molecular glue activity across diverse cellular contexts and whether tissue-specific isoform expression can be exploited therapeutically.

## Conclusions

In this study, DCAF11 isoforms 1 and 2 are found to assemble similarly into CRL4 complexes and exhibit largely overlapping endogenous substrate profiles. In contrast, they differ in their compatibility with small-molecule degraders, with covalent PROTACs engaging both isoforms and a non-covalent molecular glue exhibiting isoform 1 selectivity. These findings reveal isoform-dependent control of induced protein degradation and highlight opportunities to exploit DCAF11 isoforms for selective therapeutic targeting.

## Author contributions

X. J. Investigation, methodology, writing – original draft, writing – review & editing; X. Z. Conceptualization, supervision, funding acquisition, writing – original draft, writing – review & editing.

## Conflicts of interest

The authors declare that they have no conflicts of interest with the contents of this article.

## Supplementary Material

CB-OLF-D6CB00119J-s001

CB-OLF-D6CB00119J-s002

CB-OLF-D6CB00119J-s003

## Data Availability

The mass spectrometry proteomics data have been deposited to the ProteomeXchange Consortium *via* the PRIDE partner repository with the dataset identifier PXD076158. Supplementary information: Supplementary Figures 1–5 and Supplementary Tables 1–2 containing the protein lists from AP–MS proteomics and global proteomics. See DOI: https://doi.org/10.1039/d6cb00119j.
