# Supplementary material for: Dissecting the functional divergence of DCAF11 isoforms in protein degradation
Source: RSC Chem Biol. 2026 Jul 10;7(9):1784–92. doi: 10.1039/d6cb00119j (PMC13373504; doi:10.1039/d6cb00119j)
Supplement: Supplementary file 1 [file CB-007-D6CB00119J-s001.pdf]

Supplementary Information (SI) for RSC Chemical Biology.
This journal is © The Royal Society of Chemistry 2026

Dissecting the Functional Divergence of DCAF11 Isoforms in Protein Degradation

Xiaokang Jin[1], Xiaoyu Zhang[1,2,3,4,5*]

[1]Department of Chemistry, Northwestern University, Evanston, Illinois 60208, United States

[2]Chemistry of Life Processes Institute, Northwestern University, Evanston, Illinois 60208, United States

[3]Robert H. Lurie Comprehensive Cancer Center, Northwestern University, Chicago, Illinois 60611, United States

[4]Center for Human Immunobiology, Northwestern University, Chicago, Illinois 60611, United States

[5]International Institute for Nanotechnology, Northwestern University, Evanston, Illinois 60208, United States

*To whom correspondence should be addressed: zhang@northwestern.edu

Supplementary Figures

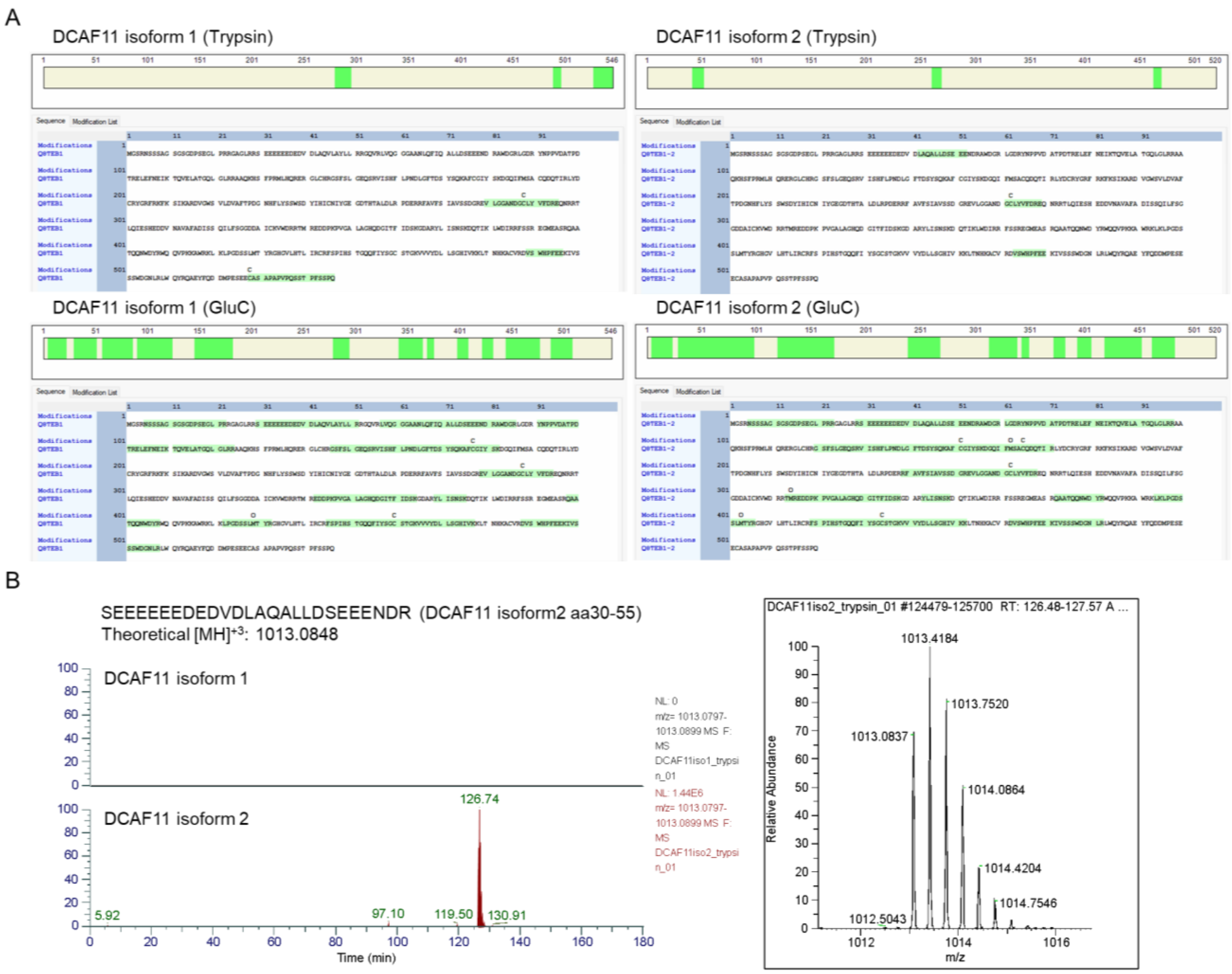

Figure S1. Validation of DCAF11 isoform-specific expression. A. Sequence coverage maps from trypsin and GluC digestion showing DCAF11 isoform 2-specific peptides detected in DCAF11 isoform 2 expressing cells but not in DCAF11 isoform 1 expressing cells. **B.** Extracted ion chromatogram (*m/z* 1013.0797-1013.0899) and MS1 spectrum of a DCAF11 isoform 2-specific peptide.

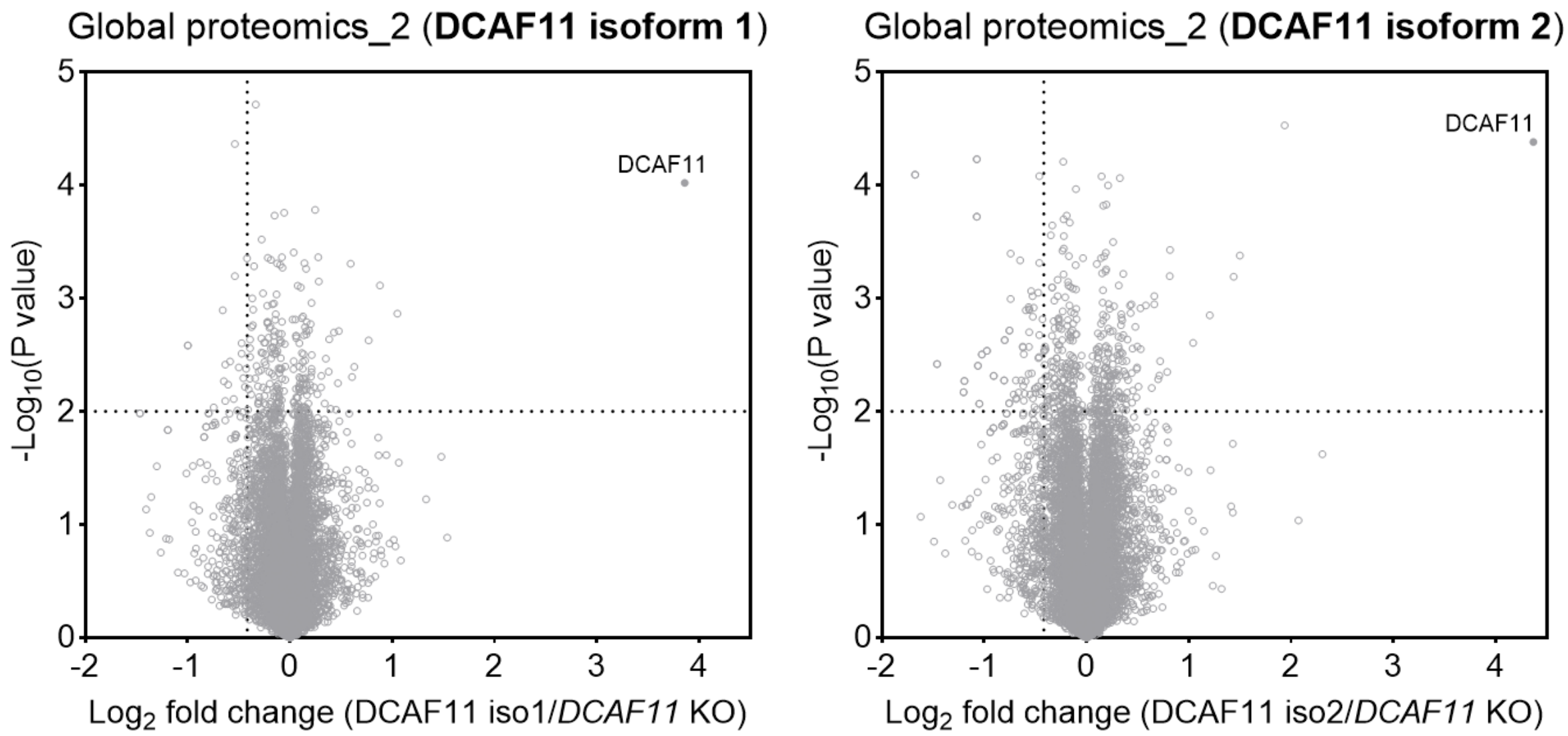

Figure S2. DCAF11 isoforms 1 and 2 regulate a shared substrate network. Volcano plot of global proteomics data comparing *DCAF11* KO cells with KO cells re-expressing DCAF11 isoforms 1 and 2 (n = 2 biologically independent samples).

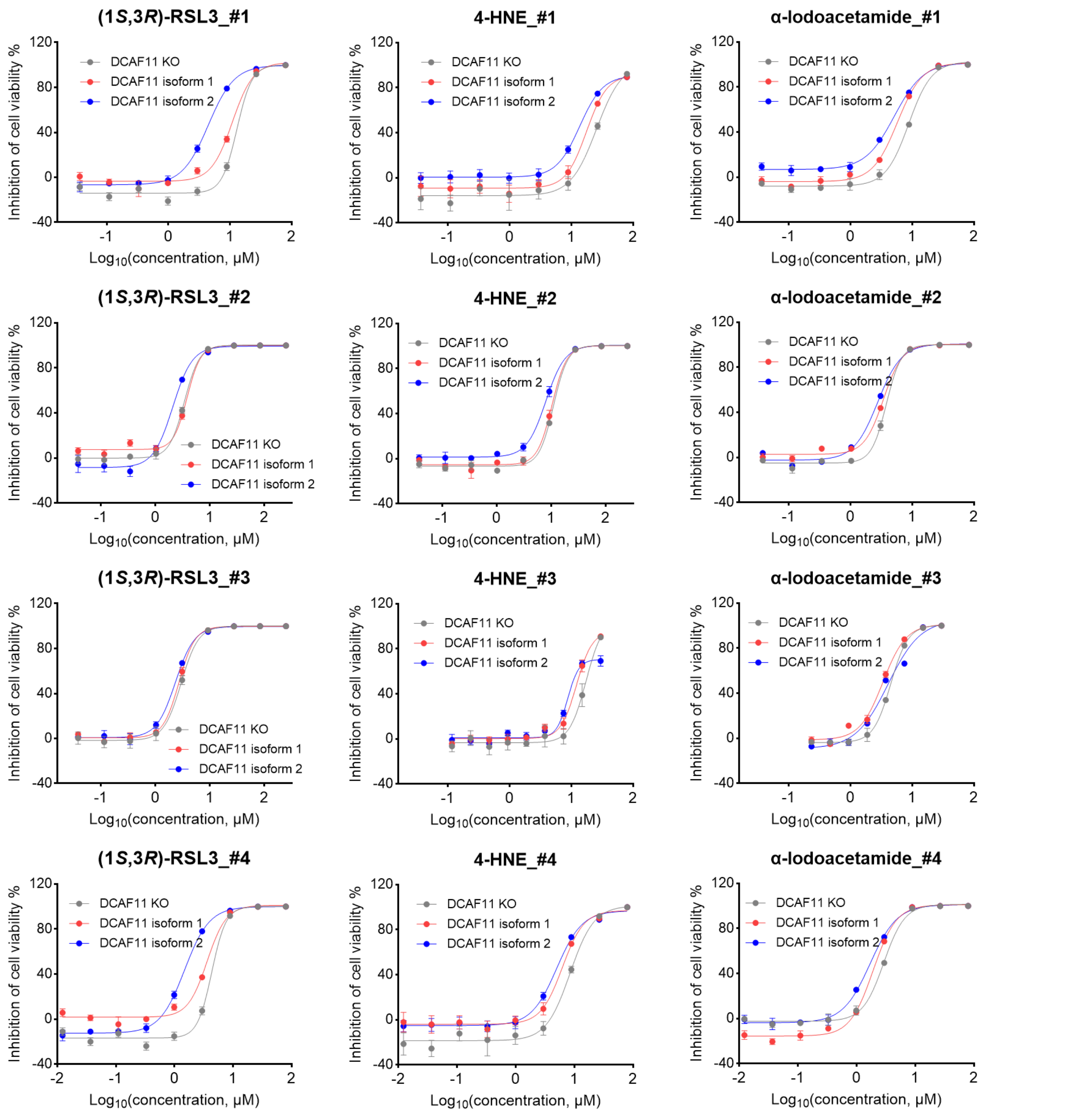

Figure S3. Dose-response curves for cell viability. Cells were treated with (1*S*,3*R*)-RSL3, 4-hydroxynonenal (4-HNE) or α-iodoacetamide in *DCAF11* KO cells and KO cells re-expressing isoform 1 or isoform 2. Data are presented as mean ± SEM (n = 3 biologically independent samples)

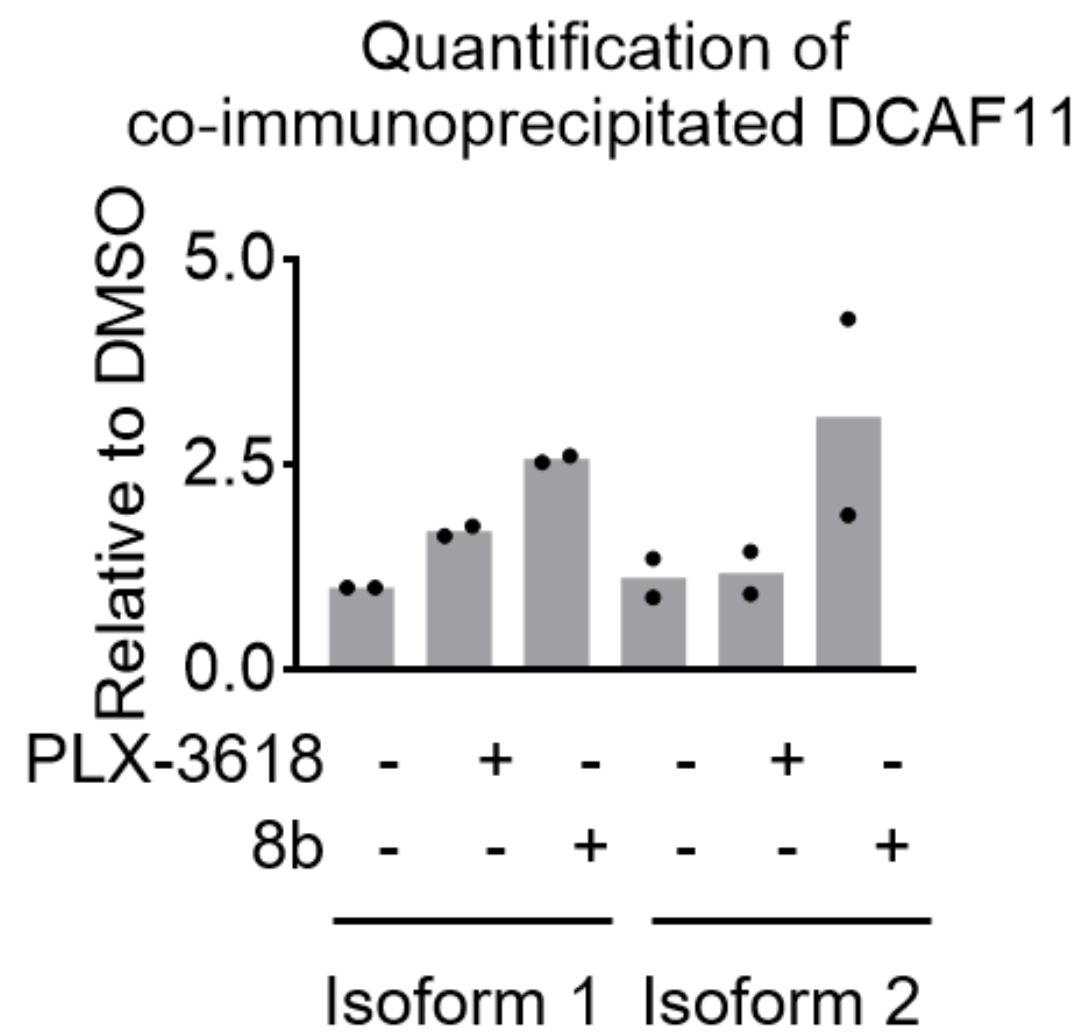

Figure S4. Quantification of immunoprecipitated HA-DCAF11 shown in Figure 4E. Data are presented as the mean values (n = 2 independent replicates).

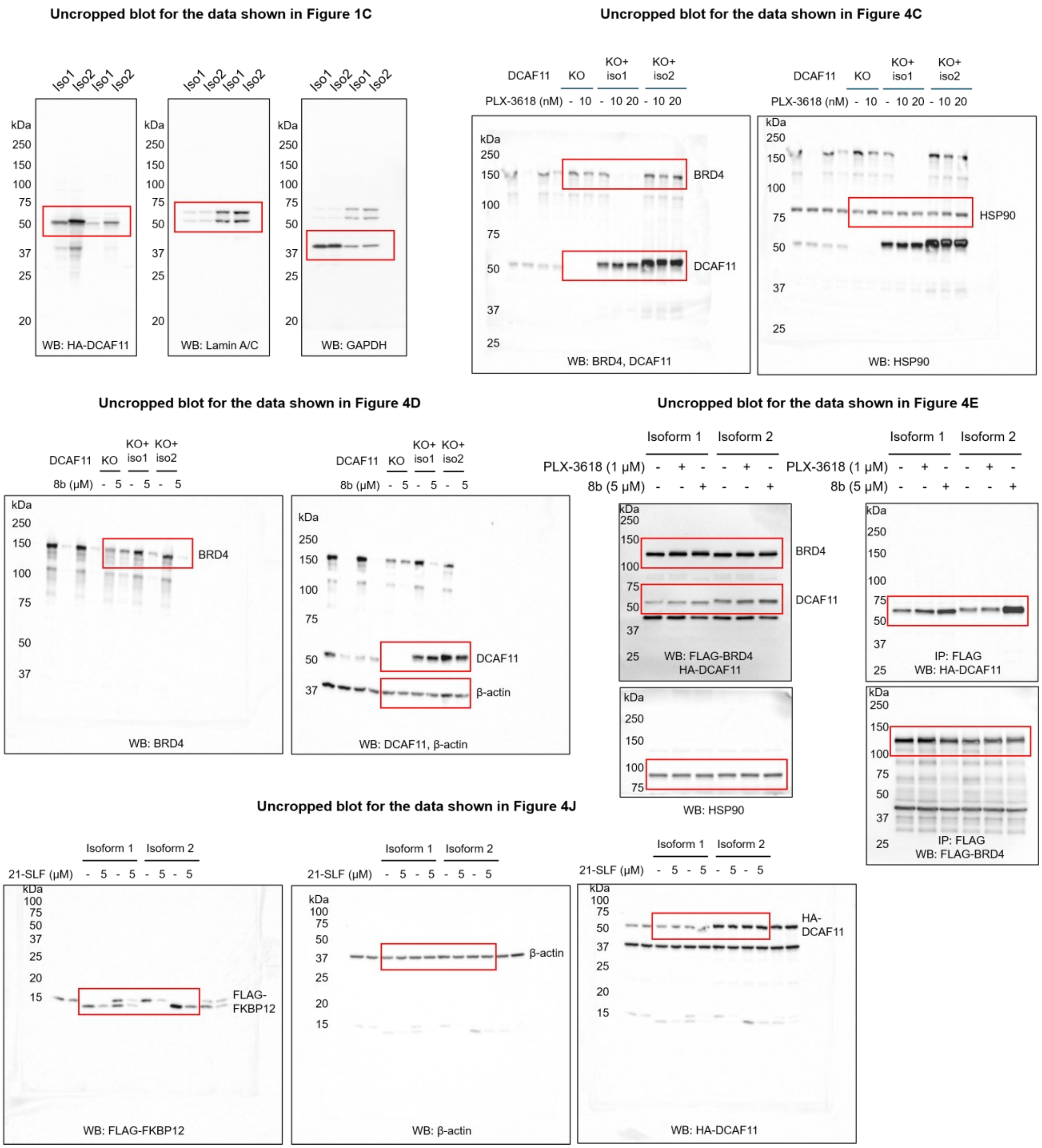

Figure S5. Uncropped blot for the data presented in the main figures.
